# Vitamin E treatment modulates steatotic and attenuates oxidative stress in a MASLD in vitro model

**DOI:** 10.1007/s11033-026-12691-0

**Published:** 2026-09-07

**Authors:** Seyedeh Kiana Teymoorian, Kosar Nouri, Mahmoud Alipour Choshali, Moustapha Hassan, Elham Rismani, Massoud Vosough, Abbas Piryaei

**Affiliations:** 1 Department of Basic Sciences and Advanced Technologies in Biology, University of Science and Culture, Tehran, Iran; 2 Department of Regenerative Medicine, Cell Science Research Center, Royan Institute for Stem Cell Biology and Technology, ACECR, 16635148 Tehran, Iran; 3 Experimental Cancer Medicine, Institution for Laboratory Medicine, Karolinska Institute, Stockholm, 141-83 Sweden; 4 Molecular Medicine Department, Biotechnology Research Center (BRC), Pasteur Institute of Iran, Tehran, Iran; 5 Department of Biology and Anatomical Sciences, School of Medicine, Shahid Beheshti University of Medical Sciences, Tehran, 1985717443 Iran

**Keywords:** MASLD, Lipid accumulation, Nrf2 signaling pathway, Oxidative stress, Vitamin E

## Abstract

**Background:**

Metabolic dysfunction-associated steatotic liver disease (MASLD) is a major chronic liver disorder and a growing global health concern driven in part by oxidative stress. Activation of antioxidant pathways, particularly the nuclear factor erythroid 2-related factor 2 (Nrf2) signaling pathway, by vitamin E represents a promising therapeutic strategy.

**Methods and Results:**

In this study, we developed a biomimetic co-culture platform to evaluate the impact of vitamin E on oxidative stress and steatosis progression in a MASLD model. The model consisted of a co-culture of Huh-7 and LX-2 cells (4:1) seeded on plates coated with 100 $\:\mathrm{μ}$g/mL liver extracellular matrix-derived hydrogel (LEMgel) from decellularized sheep liver. MASLD was induced by the co-culture treatment with Oleic acid (330 µM) and Palmitic acid (165 µM) for 2 days. Subsequently, cells were treated with 100 $\:\mathrm{μM}$ vitamin E for 4 days. Oil Red O staining and gene expression analysis of *CD36*, *SREBP-1c*, and *CPT-1* confirmed successful steatosis induction and transcriptome alterations consistent with a MASLD phenotype. Vitamin E treatment significantly improved cell viability, reduced intracellular lipid accumulation, and downregulated *CPT-1*, *NOX4*, *SREBP-1c*, *CD36*, and *PPAR*$\:\mathrm{{\gamma}}$, while upregulating *HO-1*,* NQO1*, *SOD*, and *GSH*, indicating enhanced antioxidant capacity. It also modulated hepatic markers (albumin, alpha-fetoprotein), suppressed LX-2 activation *via* TGF-β inhibition, activated Nrf2 signaling, and induced expression of downstream target genes, including the antioxidant/carboxylesterase gene CES1.

**Conclusions:**

Overall, vitamin E attenuated oxidative stress and steatosis, and the biomimetic platform provides a physiologically relevant model for MASLD pathogenesis and drug screening.

**Supplementary Information:**

The online version contains supplementary material available at https://doi.org/10.1007/s11033-026-12691-0.

## Introduction

Metabolic dysfunction-associated steatotic liver disease (MASLD) is one of the most prevalent chronic liver diseases, affecting approximately 30–38% of adults worldwide, with projections suggesting an increase beyond 55% by 2040–2045 [1]. The highest prevalence has been reported in Latin America, East/South Asia, and the Middle East/North Africa (MENA) region [2]. MASLD represents a broad disease spectrum that progresses from simple steatosis to metabolic dysfunction-associated steatohepatitis (MASH), fibrosis, and cirrhosis, which ultimately could lead to hepatocellular carcinoma (HCC). Moreover, it is one of the leading causes of liver transplantation [3, 4]. Several metabolic and lifestyle-related factors are associated with MASLD, including type 2 diabetes mellitus, obesity, insulin resistance, metabolic disorders, cardiovascular diseases, and sedentary behavior [5]. The development and severity of advanced stages of MASLD are closely linked to modifiable dietary risks, where high cholesterol intake, Westernized diets, and the synergistic damage caused by fructose and alcohol consumption significantly exacerbate hepatic injury and fibrogenesis [6–9].

The core of liver cell interactions is primarily among hepatocytes, liver-resident macrophages (Kupffer cells, KCs), and hepatic stellate cells (HSCs). Lipid accumulation in hepatocytes leads to their injury and subsequent activation of KCs, which promote inflammatory signaling [10]. The inflammation triggers HSCs activation, during which they transdifferentiate into myofibroblast-like cells. Activated HSCs are characterized by increased production of reactive oxygen species (ROS), extracellular matrix (ECM) components, and transforming growth factor-beta (TGF-$\:\mathrm{{\beta}}$) [10]. Since the current study specifically focused on hepatocellular lipid dysregulation, oxidative stress, and direct hepatocyte-HSC interactions, we employed hepatocyte-like cells (Huh-7) and HSCs (LX-2) to establish the MASLD model. This simplified co-culture system, supported by liver-derived ECM, provides a controlled platform to dissect Nrf2-mediated redox signaling and hepatocyte-HSC crosstalk in the absence of additional immune cell components.

Since oxidative stress is one of the most significant factors in the transition of MASLD toward fibrotic MASH, redox pathways are considered potential therapeutic targets. Excessive hepatic lipid accumulation enhances mitochondrial $\:\beta\:$-oxidation, leading to increased ROS production, impaired antioxidant defenses, endoplasmic reticulum (ER) stress, and fibrosis [11, 12]. Oxidative stress is also closely associated with insulin resistance, inflammation, and HSCs activation [13]. Major antioxidant defense systems include the nuclear factor erythroid 2-related factor 2 (Nrf2) signaling pathway, antioxidant enzymes, glutathione, the thiol-redox system, and the regulation of iron metabolism and ferroptosis [13, 14]. Therefore, targeting these pathways may represent a promising therapeutic option.

Vitamin E is a lipid-soluble antioxidant that scavenges ROS, modulates inflammation, and regulates cell death pathways [15]. It neutralizes lipid radicals and inhibits lipid peroxidation, enhances antioxidant enzyme activity, such as superoxide dismutase (SOD), catalase, and glutathione peroxidase, and downregulates lipogenic gene expression [15, 16]. The potential relationship between vitamin E and the Nrf2 signaling pathway remains under investigation. Nrf2 is a master regulator of the cellular defense against oxidative stress, functioning as a transcription factor that induces the expression of antioxidant proteins, phase I/II detoxification enzymes, and regulators of mitochondrial function and intermediary metabolism [17, 18].

In order to study the effectiveness of a drug, a biomimetic and reliable model was required. In vivo animal models are widely used, but they do not fully replicate human MASLD due to interspecies differences, as well as ethical and cost-related limitations [19]. On the other hand, conventional two-dimensional (2D) monoculture systems are unable to replicate the complexity of hepatic cellular interactions due to their limited cellular heterogeneity and lack of ECM components. Consequently, they fail to accurately recapitulate drug responsiveness and disease progression [20–22].

The limited evidence regarding the effects of vitamin E on MASLD through Nrf2 signaling highlights a critical knowledge gap. Previous studies have mainly evaluated vitamin E in monoculture systems or animal models, and the effect of vitamin E in an ECM-supported multicellular MASLD model remains unknown. Therefore, in the present study, we benefited from a feasible 2D biomimetic liver model to study the hepatoprotective effect of vitamin E in an in vitro MASLD model. This model is composed of a co-culture system incorporating hepatocytes and hepatic stellate cell surrogates seeded on a physiologically relevant liver ECM. The results showed that vitamin E enhanced the antioxidant system, decreased lipid accumulation, and increased hepatic-specific functions, being accompanied by modulation of the Nrf2 signaling pathway.

## Materials and methods

### Ethical approval statement

All experimental procedures were performed in compliance with the ethical standards of the Ethics Committee of the University of Science and Culture (IR.ACECR.USC.REC.1404.101), Tehran, Iran.

### Cell culture and co-culture configuration

Two cell lines were used in this study: Huh-7 cells (RRID: CVCL_0336) and LX-2 cells (RRID: CVCL_5792), obtained from Royan Stem Cell Bank (Iran). The cell lines were maintained in high-glucose Dulbecco’s Modified Eagle Medium (HG-DMEM, 11995-040 Gibco, Thermo Fisher Scientific, Waltham, MA, USA) supplemented with fetal bovine serum (FBS, 16140-071, Gibco, Thermo Fisher Scientific, Waltham, MA, USA) at 10% for Huh-7 and 2% for LX-2, 1% of penicillin/streptomycin (Pen/Strep, 15070-063, Gibco, Thermo Fisher Scientific, Waltham, MA, USA), 1% non-essential amino acids (11140-035, Gibco, Thermo Fisher Scientific, Waltham, MA, USA), and 1% GlutaMAX™ (35050-038, Gibco, Thermo Fisher Scientific, Waltham, MA, USA) under standard culture conditions (37 °C and 5% CO_2_ in a humidified atmosphere).

### Preparation and surface coating of liver ECM hydrogel

Liver-derived ECM hydrogel (LEMgel) was prepared based on our previously reported protocols, with minor modification [23]. In summary, sheep liver was first cut into −2 mm-thick small pieces and rinsed with distilled water to eliminate any residual blood. The samples were then immersed in 1% sodium dodecyl sulfate (SDS, 436143, Sigma-Aldrich, St. Louis, MO, USA) solution and stirred continuously for 36 h at 4 °C. Following rinsing with double-distilled water (ddwater), the samples were immersed in 1% Triton X-100 (108643, Merck, Burlington, MA, USA) for one hour and subsequently washed with ddwater at short intervals. After lyophilizing using a Christ lyophilizer (Alpha 1–2 LDplus, Osterode am Haer, Germany), it was dissolved using 1% pepsin (P6887, Merck, Burlington, MA, USA) in 0.5 M acetic acid (Sigma-Aldrich, St. Louis, MO, USA) for 48 h on the stirrer at 4 °C to achieve a 10 mg/mL LEMgel. The pH was neutralized by gradually adding NaOH (pH −7.4).

The stock solution was diluted with basal medium to achieve a final coating concentration of 100 $\:\mathrm{μg}\mathrm{/mL}$ [24]. The LEMgel solution (100 $\:\mathrm{μg}\mathrm{/mL}$) was applied to culture wells and incubated at 37 °C for 90 min. Wells were then washed with phosphate-buffered saline (PBS) and allowed to dry under sterile conditions. Coating efficiency was verified by phase-contrast microscopic observation (Olympus Corporation, Tokyo, Japan).

### MASLD model generation and characterization

For the biomimetic co-culture platform, Huh-7 and LX-2 cells were seeded at a ratio of 4:1 in HG-DMEM supplemented with FBS 2%, 1% Pen/Strep, 1% non-essential amino acids, and 1% GlutaMAX™ [25] on the LEMgel-coated culture dishes. Steatosis was induced using a free fatty acids (FFAs) mixture according to an established protocol with slight modifications [26]. The induction medium contained 330 µM of Oleic Acid, 165 µM Palmitic Acid (final concentrations), complexed to 1% fatty acid-free Bovine Albumin Serum (A8806, Sigma-Aldrich, St. Louis, MO, USA) (FAF-BSA). In the FFAs group, cells were exposed to medium containing FFAs for 2 days to induce MASLD, followed by culture in HG-DMEM for up to 6 days, with half-medium replacement every 2 days. In the control group, the co-culture was maintained under identical conditions without FFAs.

### Preparation of free fatty acids solutions

Palmitic acid (P0500, Sigma-Aldrich, St. Louis, MO, USA) was dissolved in 0.1 M NaOH and incubated at 70 °C for 10–15 min until a clear solution was obtained. The PA solution was added dropwise to a pre-warmed 0.9% NaCl containing 10% (w/v) fatty acid-free BSA, and incubated at 37 °C for 30 min to form a stable complex. Oleic acid (O1383, Sigma-Aldrich, St. Louis, MO, USA), which is liquid at room temperature, was directly added to the BSA solution and stirred for 30 min to prepare a mixed FFA–BSA stock solution. The resulting FFA-BSA stock was subsequently diluted with culture medium to obtain final concentrations of 330 µM of Oleic Acid, 165 µM Palmitic Acid. To enhance solubility, 70% ethanol was used during stock preparation, ensuring that the final ethanol concentration did not exceed 0.05% to avoid cytotoxicity [27].

### Preparation of Vitamin E

Initially, a 2000 X concentration stock solution of 400 mM vitamin E (T1157, Sigma-Aldrich, St. Louis, MO, USA) was prepared in 95% ethanol. Next, a 40 mM intermediate concentration was prepared by dissolving the stock solution in basal medium of HG-DMEM. Furthermore, to reach the desired working concentrations of 20, 50, 100, 150, and 200 $\:\mathrm{μM}$, an intermediate concentration was diluted in supplemented HG-DMEM. The final ethanol concentration did not exceed 0.05% (v/v) [22, 28, 29].

### Vitamin E dose escalation and treatment on the MASLD model

In the vitamin E group, cells were exposed to FFAs for 2 initial days and then treated with vitamin E for 2 days. On day 2, the vitamin E-containing half medium was refreshed to maintain a constant concentration of vitamin E. On the other hand, the FFAs group was treated with FFAs for 2 days, followed by replacement with HG-DMEM for up to 2 days. The control group was cultured with HG-DMEM throughout the experiment.

### Cell viability assessment

To evaluate the biocompatibility of the LEMgel and the cell viability in the experimental groups, the 3-(4,5-dimethylthiazol-2-yl)-5-(3-carboxymethoxyphenyl)-2-(4-sulfophenyl)–2 H-tetrazolium (MTS, G1111, Promega, Madison, WI, USA) assay was performed. To assess LEMgel biocompatibility, the LEMgel-coated 96-well plates were seeded with a co-culture of 4 × 10^3^ Huh-7 cells and 1 × 10^3^ LX-2 cells per well with 100 $\:\mathrm{μL}$ culture medium. The control group was the same culture on neat plates. After 1 day, 20 $\:\mathrm{μl}$ of MTS reagent was added and incubated for 1 h. Absorbance was measured at 490 nm using a microplate reader (51118650, Multiskan Spectrum, Thermo Fisher Scientific Waltham, MA, USA) [27].

### Intracellular lipid accumulation analysis

Intracellular lipid accumulation was evaluated to confirm the establishment of MASLD and the effect of vitamin E on the model using Oil Red O (O0625, Sigma-Aldrich, St. Louis, MO, USA) or Nile Red (19123, Sigma- Aldrich, St. Louis, MO, USA). Oil Red O stains neutral lipids, such as triglycerides, red. Nile Red produces a range of fluorescent colors from orange-yellow gold for triglycerides to dark red for membrane phospholipids [26].

For this purpose, at the target time points, the cells were fixed with 4% formaldehyde for 30 min. For Oil Red O staining, the cells were incubated with 0.3% Oil Red O solution for 15 min. For quantification, after washing, the dye was extracted from the cells using 200 $\:\mathrm{μl}$ absolute isopropanol and absorbance was measured at 500 nm [30]. Then, the measured OD was normalized to the cell numbers of its corresponding well.

Nile Red staining was performed for furthered verifications and quantification. For this staining, fixed cells were incubated with 10 $\:\mathrm{μM}$ dye in methanol at room temperature for 10 min. Plates were washed with PBS and micrographs were acquired by an Olympus IX71 fluorescent microscope (Olympus Corporation, Tokyo, Japan). The mean fluorescent intensity for the orange-yellow gold spectrum of at least 30 randomly selected high-power microscopic fields per group was quantified using ImageJ software [27].

### Gene expression analysis by qPCR

For gene expression analysis, total RNA was extracted using the TRIzol (15596018, Thermo Fisher Scientific, Waltham, MA, USA)/chloroform method according to the manufacturer’s guidelines. RNA integrity was confirmed by gel electrophoresis, and concentration was measured spectrophotometrically (Biochrom WPA Biowave ii). Complementary DNA (cDNA) synthesis was performed using a reverse transcriptase kit (Easy cDNA Ultra-TM Synthesis Kit, Parstous, Iran). Quantitative PCR (qPCR) was conducted using SYBER Green Master Mix (A3254022, Ampliqon, Denmark) on an Applied Biosystem StepOne real-time PCR system. Gene expression was normalized to glyceraldehyde-3-phosphate dehydrogenase (*GAPDH*) and compared against the corresponding control group expression values. The data were analyzed using the comparative CT method (2^−^$\:\mathrm{∆∆}\mathrm{Ct}$) [27]. Primer sequences are listed in Supplementary Table 1.

### Quantification of secreted proteins by ELISA

To evaluate the MASLD model establishment and the impact of vitamin E on the functional profile of the MASLD model, enzyme-linked immunosorbent assay (ELISA) was performed using commercial kits. Cells were initially seeded at 187,500 cells/well at a Huh-7: LX-2 ratio of 4:1, and incubated with FFAs for 2 days. After 4 days of vitamin E treatment, conditioned media were collected and centrifuged to remove cell debris. All values were normalized to the total protein content of the corresponding well. Albumin (ALB), alpha-fetoprotein (AFP), superoxide dismutase (SOD), and TGF-$\:\mathrm{{\beta}}$ were quantified using ELISA kits, whereas glutathione (GSH) was measured by a colorimetric assay kit, following the manufacturer’s instructions. Kits are provided in Supplementary Table 2.

### Protein expression analysis by Western blot

Western blot analysis was performed to assess the expression of Nrf2 (main transcription factor in the Nrf2 signaling pathway) and CES1 (downstream protein of the Nrf2 signaling pathway). Cells were seeded at 250,000/well with a Huh-7: LX-2 ratio of 4:1. After 2 days of FFAs treatment, vitamin E exposure was initiated and continued for 2 days. Cells were subsequently harvested on day 4. This earlier endpoint was selected to evaluate the early transcriptional response of Nrf2 and CES1 to vitamin E, prior to later phenotypic changes assessed at day 6.

Briefly, samples were lysed in Tris-based buffer (pH − 8.0) containing detergents and protease inhibitors, followed by centrifugation (13,200 g, 10 min, 4 °C to collect the protein-containing supernatant. Protein concentration was determined using the Bradford method based on Coomassie Brilliant Blue G-250 dye binding at 630 nm, using a BSA standard curve as reference Marion M. Bradford. Equal amounts of protein were denatured in 3× sample buffer and separated by SDS-PAGE (10% resolving gel). Proteins were transferred onto PVDF membranes by wet transfer (120 V, 90 min), followed by blocking with 2% non-fat milk in TBS-T. Membranes were incubated at 4 °C with primary antibodies (Nrf2: A11 sc-365949; 1:300, CES1: A 10 sc-390999; 1:300, and $\:\mathrm{{\beta}}$-actin: sc-365249; 1:300, Santa Cruz Biotechnology, Inc. Dallas, TX, USA) overnight. After washing, membranes were incubated for 1 h with HRP-conjugated secondary antibodies: goat anti-mouse IgG-HRP (sc-516102, 1:1000) and goat anti-rabbit IgG-HRP (sc-2357, 1:1000). Detection was performed using X-ray film exposure. Protein levels were normalized to β-actin. Band relative density was quantified using ImageJ software.

### Statistical analysis

All data were analyzed using GraphPad Prism Software (V.10.3.1, GraphPad Software Inc., San Diego, CA, USA). To evaluate the normal distribution of the data, the Shapiro-Wilk test was used. The comparisons between two groups were carried out using unpaired two-tailed Student’s t-test, while comparisons between more than two groups were performed using one-way ANOVA. Data are presented as mean $\:\pm\:$ standard deviation (SD). *P*-values < 0.05 were considered statistically significant.

## Results

### Establishment and characterization of the in vitro MASLD model

**Fig. 1:**
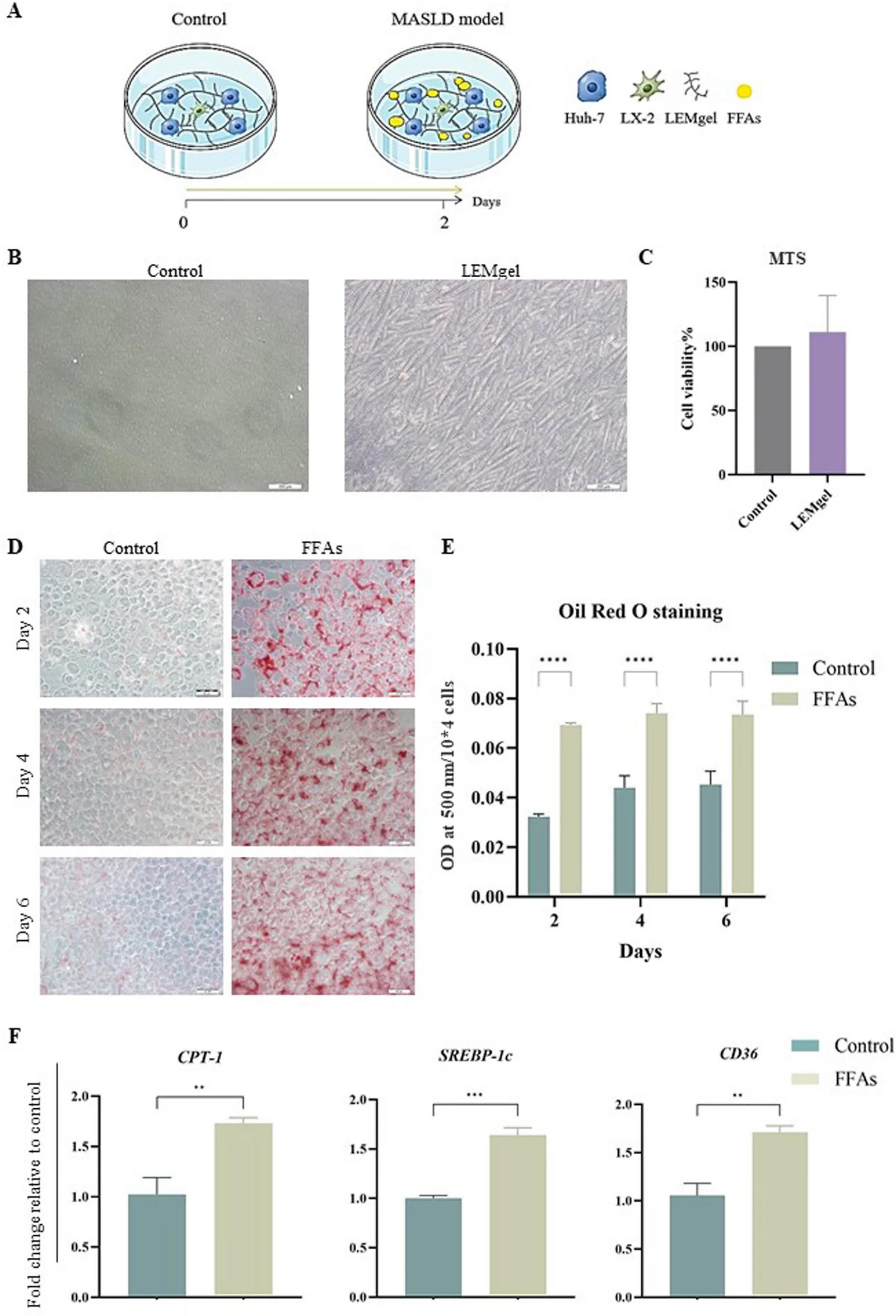
MASLD model establishment assessment. (**A**) Timeline of MASLD model establishment. (**B**) Microscopical images confirmed the ECM coating on the surface of culture plate. (**C**) MTS test indicated no significant difference between control group and the ECM-coated group, which suggested that ECM had no cytotoxicity effects on cells. (**D**) To evaluate the stability of MASLD model, cells were treated with 33 mM OA and 16.5 mM PA for 2 days, and after this time, the media was refreshed with DMEM 2% FBS every 2 days for 6 days. Oil Red O staining was performed for 2, 4, and 6 days. (**E**) Each state showed significant differences between control and FFAs group; Oil red O staining was quantified, and each day indicated significant lipid accumulation. (**F**) qPCR determined the expression of SREBP-1c. CD36 and CPT-1 were upregulated in FFAs group compared with control after 2 days of FFAs treatment. Data are shown as mean ± SD. *p < 0.05, **p < 0.01, ***p < 0.001, ****p < 0.0001

The MASLD model was produced using co-culture of Huh-7 and LX-2 cell lines (Fig. 1A), seeded on LEMgel-coated plates. To evaluate successful LEMgel coating, phase contrast micrographs were taken (Fig. 1B). Phase-contrast microscopy indicated a fiber structure, most likely composed of collagen. MTS assay demonstrated no significant differences between the control and LEMgel-coated groups, which implied that the LEMgel coating was biocompatible with no cytotoxic effect (Fig. 1C).

The MASLD model was induced by the exposure of co-culture cell to FFAs for 2 days. Compared to the control group, representative micrographs and quantification of Oil Red O staining demonstrated significant intracellular lipid accumulation in the FFAs group, which was stable over 6 days of culture (Fig. 1D, E). Moreover, gene expression assessments revealed that fatty acid $\:\mathrm{{\beta}}$-oxidation, carnitine palmitoyltransferase 1 (*CPT-1)*,* de no vo* lipogenesis gene, sterol regulatory element binding transcription factor 1 isoform 1c (*SREBP-1c)*, and fatty acid translocase gene, cluster of differentiation 36 (*CD36)* significantly upregulated on the day 2 following the 2 initial days of FFAs exposure (*P* < 0.01, *P* < 0.001, and *P* < 0.01) (Fig. 1F). Taken together, these data confirmed successful MASLD induction in the in vitro model.

### Vitamin E dose escalation

**Fig. 2:**
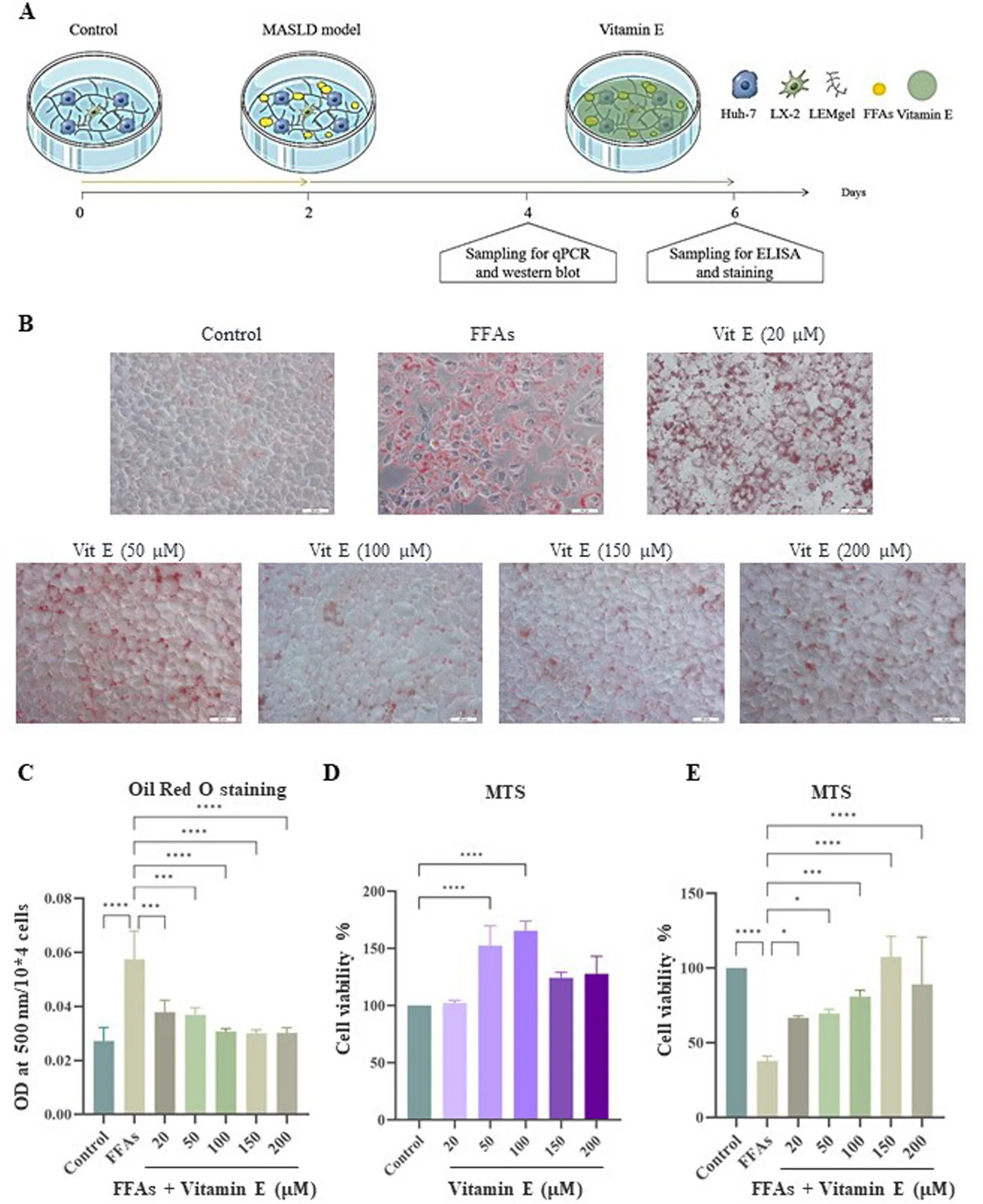
Dose escalation of vitamin E on control, FFAs, and Vitamin E groups. (**A**) Timeline of vitamin E treatment and sampling. (**B**) Oil Red O staining of vitamin E dose escalation was conducted, and the results showed that all the selected doses reduced the lipid accumulation after 4 days of treatment. (**C**) The quantification of Oil Red O staining indicated that 100, 150, and 200 $\:\mathrm{μM}$ of vitamin E has the same reduction of lipid accumulation, so the lowest concentration with high efficacy was selected, which was 100 $\:\mathrm{μM}$. (**D**) Based on the MTS outcomes, vitamin E increased the cell viability and activity in control cells. (**E**) MTS results indicated that vitamin E treatment increased the cell viability in comparison with FFAs group. Data are shown as mean ± SD. *p < 0.05, **p < 0.01, ***p < 0.001, ****p < 0.0001

To determine the optimal concentration of vitamin E for treatment of the MASLD model, Oil Red O staining was performed to visualize and quantify the lipid accumulation. Figure 2A shows the timeline of sampling. The data showed all the tested concentrations of vitamin E (20, 50, 100, 150, and 200 $\:\mathrm{μM}$) significantly reduced intracellular lipid accumulation in the MASLD model. The concentrations ranging from 100 to 200 $\:\mathrm{μM}$ showed the most pronounced effects, reaching about half compared to the FFAs group (*P* < 0.0001) with no significant difference to the control group (Fig. 2B, C).

MTS assay indicated that vitamin E significantly increased cell proliferation in the control group at 50 and 100 $\:\mathrm{μM}$ concentrations (*P* < 0.0001) (Fig. 2D). Moreover, the cell viability was significantly improved in all doses of vitamin E treatment in FFAs groups (Fig. 2E).

The concentration of 100 $\:\mathrm{μM}$ of vitamin E was selected as the optimal dose, because it was the lowest concentration that markedly reduced FFAs-induced lipid accumulation, while remaining within a non-cytotoxic range as determined by the MTS assay.

On the other hand, since Oil Red O staining demonstrated no significant effect of vitamin E`s vehicle on lipid accumulation, the vehicle control was excluded for the rest of the study (Fig. S1).

### Vitamin E treatment modulates gene expression in the in vitro MASLD model

Following 2 initial days FFAs exposure, gene expression data on day 6 showed that, compared to the control group, the transcripts for the mitochondrial fatty acid transport gene *CPT-1* and the ROS-generating enzyme (NADPH oxidase 4, *NOX4*) were increased following FFAs exposure (both *P* < 0.001). However, compared to the FFAs group, expression of these genes was significantly (*P* < 0.05 and *P* < 0.001) downregulated in the vitamin E-treated group. The antioxidant enzyme genes, including heme oxygenase 1 (*HO-1*) and NAD(P)H quinone dehydrogenase 1 (*NQO1)*, were significantly downregulated in the FFAs group (*P* < 0.01 and *P* < 0.001, respectively), whereas vitamin E treatment upregulated both genes (*P* < 0.05) compared to the FFAs group.

**Fig. 3:**
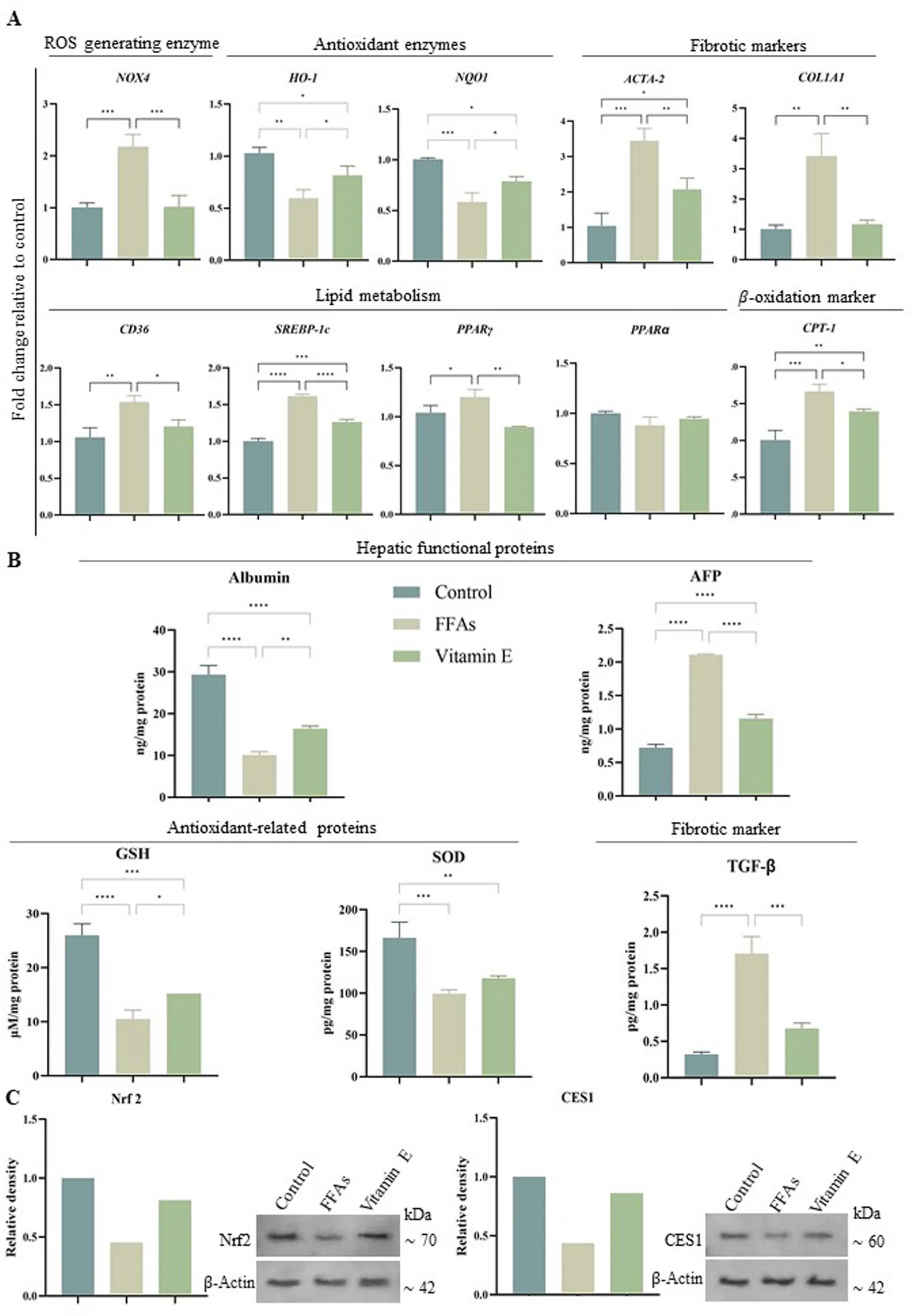
The impact of vitamin E on gene and protein expression. (**A**) The qPCR results indicated vitamin E causes overexpression of antioxidant genes, while downregulating genes associated with mitochondrial function, lipid metabolism, and fibrotic markers. (**B**) Based on the results of ELISA tests, albumin has been decreased in FFAs and increased in vitamin E group. On the other hand, the AFP has been increased in FFAs and decreased in vitamin E group, which suggested that vitamin E improved the function of hepatocytes. GSH and SOD have been decreased in FFAs; vitamin E increased GSH, but did not elevate SOD. TGF-β, as a factor of LX-2 cells activation, has been significantly upregulated in FFAs group, and vitamin E downregulated its expression. (**C**) Western blot of Nrf2 and CES1 showed that FFAs can reduce their expression, and vitamin E has an activating effect on their expression. Data are shown as mean ± SD. *p < 0.05, **p < 0.01, ***p < 0.001, ****p < 0.0001

Evaluation of the two fibrotic markers, smooth muscle alpha-2 actin (*ACTA-2*) and collagen type I alpha 1 chain (*COL1A1)*, demonstrated their induced expression in the FFAs group (*P* < 0.001 and *P* < 0.01, respectively), while vitamin E treatment significantly decreased their expressions, compared to the FFAs group (*P* < 0.01 and *P* < 0.01, respectively) (Fig. 3A).

Considering the lipid metabolism-related genes’ transcripts, including *CD36*, *SREBP-1c*, and peroxisome proliferator-activated receptor gamma (*PPAR*$\:\mathrm{{\gamma}}$*)* we found a significant upregulation in the FFAs group (*P* < 0.01, *P* < 0.0001, and *P <* 0.05, respectively) in comparison to the control ones. Moreover, compared to the FFAs group was subsequently downregulated following vitamin E treatment (*P* < 0.05, *P* < 0.0001, and *P* < 0.01, respectively). However, no significant change was observed in the transcripts for adipogenesis transcription factor (peroxisome proliferator-activated receptor alpha, *PPAR*$\:\mathrm{{\alpha}}$) among the experimental groups (Fig. 3A). Taken together, these data indicated that vitamin E treatment enhanced cellular antioxidant response, as evidenced by an increase in antioxidant gene expression, and lipogenesis, and enhanced lipolysis in the MASLD model.

### Vitamin E improved the secretion of hepatic and antioxidant proteins and decreased fibrotic biomarkers

Evaluation of the secreted protein profile was conducted on day 6, following 2 initial days FFAs exposure and 4 days of vitamin E treatment, using ELISA. Compared to the control group, albumin (ALB) as an indicator of the mature hepatic cells’ function, significantly (*P* < 0.0001) decreased in the FFAs group, while vitamin E treatment considerably (*P* < 0.01) elevated its secretion in comparison to the FFAs group. Conversely, AFP, a biomarker of immature hepatocytes, significantly (*P* < 0.0001) increased in the FFAs group compared to the control, however dramatically (*P* < 0.0001) lowered to half after treatment with vitamin E.

In comparison to the control group, antioxidant proteins, GSH and SOD, both showed a marked (*P* < 0.0001 and *P* < 0.001, respectively) reduction in secretion in the FFAs group. Although vitamin E treatment significantly (*P* < 0.05) increased the GSH protein, it did not alter SOD compared to the FFAs group.

The fibrotic biomarker TGF-$\:\mathrm{{\beta}}$, which activates LX-2 cells, demonstrated a pronounced (*P* < 0.0001) increase in the FFAs group compared to the control group; and vitamin E treatment lessened this factor considerably (*P* < 0.001) in comparison to the FFAs group (Fig. 3B). Taken together, these data showed that vitamin E improved hepatic function and decreased oxidative stress and liver fibrosis in the MASLD model.

### Vitamin E enhanced cellular protection against lipid toxicity in the MASLD model

Protein expression of Nrf2, which induces antioxidant enzymes, and CES1, a lipid metabolism enzyme, were evaluated using Western blot assessment. Data showed that in comparison to the control group, both of the proteins decreased by half in the MASLD model. However, vitamin E treatment restored protein expression to approximately control levels (Fig. 3C). Thus, these results demonstrated that vitamin E protected the cells against oxidative stress and lipid accumulation.

### Vitamin E reduces intracellular lipid accumulation in the MASLD model

**Fig. 4:**
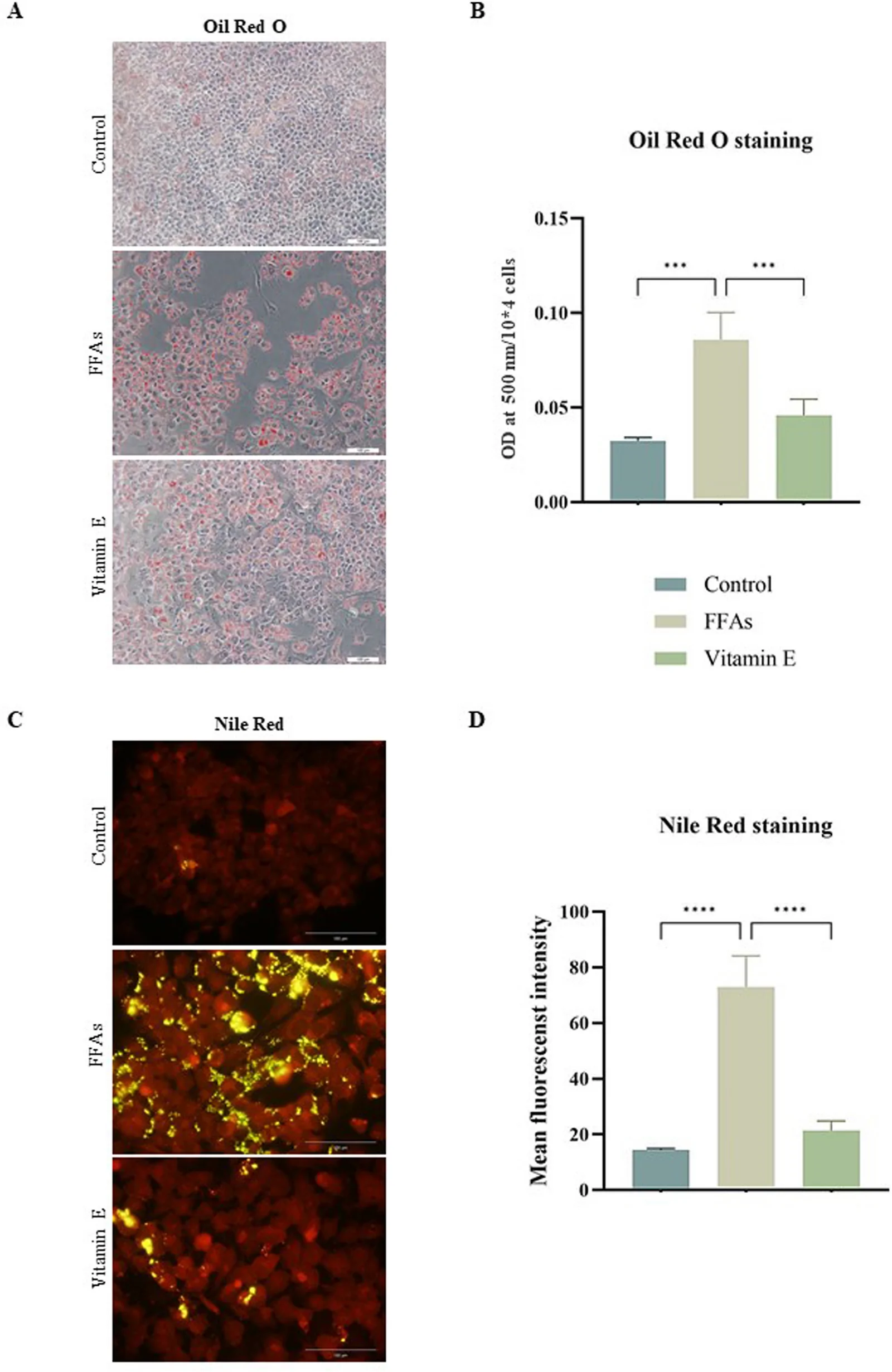
Lipid accumulation analysis following vitamin E treatment. (**A**) According to the microscopic figures of Oil red O staining, vitamin E has the capacity to reduce lipid accumulation in the MASLD model after 4 days. (**B**) The quantification of Oil red O staining confirmed the qualitative images. (**C**) Nile red staining confirmed that vitamin E can reduce the intracellular neutral lipid accumulation after 4 days of treatment. (**D**) The mean fluorescent intensity of Nile red staining suggested decreased lipid accumulation in vitamin E group compared to FFAs. Data are shown as mean ± SD. *p < 0.05, **p < 0.01, ***p < 0.001, ****p < 0.0001

To confirm the impact of vitamin E on lipid accumulation in the MASLD model, Oil Red O and Nile Red staining were performed after 4 days of treatment. Micrographs of Oil Red O staining showed marked lipid accumulation in the FFAs group, while vitamin E reduced the intracellular lipid deposition in the MASLD model (Fig. 4A). Quantification analysis of Oil Red O staining further corroborated these findings, demonstrating a significant (*P* < 0.001) decrease in lipid accumulation in the vitamin E-treated group compared to the FFAs group (Fig. 4B).

In comparison to the control group, fluorescent micrographs from Nile Red staining indicated excess lipid content in the FFAs group which attenuated by vitamin E treatment (Fig. 4C). Quantification of Nile Red mean fluorescent intensity confirmed a significant (*P* < 0.0001) reduction in intracellular lipid content following vitamin E treatment in the MASLD model (Fig. 4D). Together, these results showed that vitamin E treatment reduced lipid accumulation in the MASLD model.

## Discussion

As the number of individuals affected by MASLD continues to rise, there is an urgent need for novel experimental platforms and innovative therapeutic approaches [1]. The lack of reliable models to investigate MASLD mechanisms and evaluate drug responses represents a major challenge, limiting disease understanding and therapy development. In addition, the absence of approved therapies for MASLD highlights the urgent need for novel drugs [31]. In this study, a biomimetic model was developed using a co-culture of Huh-7 and LX-2 cells on a LEMgel-coated plate. MASLD was induced by Oleic acid and Palmitic acid, and the in vitro model expressed some key features of the disease. Subsequently, our results indicated that vitamin E treatment may exert its protective effect against oxidative stress, modulate lipid metabolism-related genes, reduce lipid content, and activate LX-2 cells in the MASLD model. Moreover, these effects appear to be mediated, suggesting that at least in part, through activation of the Nrf2 signaling pathway. However, because Nrf2/CES1 protein data were obtained as supportive semi-quantitative evidence and no Nrf2 inhibition/knockdown experiment was performed, a causal role for Nrf2 cannot be concluded.

In this study, by co-culturing Huh-7 and Lx-2 cells on LEMgel substrate, we tried to develop a more reliable biomimetic in vitro MASLD model. Conventional monocultures of hepatocytes cannot accurately reproduce the model, due to a lack of interactions between hepatocytes and non-parenchymal cells [31]. Therefore, co-culture of liver parenchymal and non-parenchymal liver cells represents an important improvement over the conventional monoculture model. Moreover, adding LEMgel to the co-culture system provides both structural and biochemical features that better resemble the native hepatic microenvironment. Saheli et al. confirmed that incorporating the liver tissue-specific hydrogel, LEMgel, into liver organoid cultures significantly improved functionality compared with conventional collagen hydrogel. LEMgel-based hydrogel demonstrated significantly higher albumin secretion and urea production, improved CYP3A4 activity, and increased expression of hepatic genes, indicating enhanced liver-specific function [23].

Importantly, the present model differs from conventional monocultures by incorporating hepatocyte-like and hepatic stellate cells within a liver-derived ECM base. Although 3D models demonstrate better response and more tissue-specific characteristics, they are often expensive, time-consuming, and most likely to have batch-to-batch variations [32]. Our model acts as intermediate platform that combines simplicity and accessibility of 2D system with key cell-cell and cell-ECM interactions characteristic of 3D cultures.

In order to induce steatosis, a mixture of FFAs was used in the experiment. FFAs exposure has been shown to induce classical features of lipotoxicity, including cellular lipid accumulation, increased oxidative stress, and reduced cell viability [16]. Consistently, our MASLD model increased cellular lipid content and decreased cell viability (*P* < 0.0001), due to a high uptake of FFAs and its cytotoxic effects. Live-dead staining (*P* < 0.01) and MTS assay confirmed that FFAs treatment increased cell death and reduced viability in NAFLD microtissues [27]. Furthermore, our results indicated significant increase in the transcripts for *CPT-1* and *SREBP-1c* genes in the MASLD model compared to the control group, showing that FFAs led to increased fatty acid β-oxidation and lipogenesis. Similarly, in 2025, Majidi et al. showed that treatment of microtissues with FFAs for 7 days led to the upregulation of these two genes [27]. Moreover, our results indicated that *CD36* expression was increased in MASLD, which shows the increased cellular uptake of long-chain fatty acids. However, Asadallahi et al. did not observe a significant difference in *CD36* expression between their NAFLD microtissue model and the control group [26]. Overall, the MASLD model recapitulated the key features of liver physiology and steatosis, which makes it an appropriate platform to study drug response for the treatment of the disease.

In the current study, vitamin E treatment was considered a therapeutic supplement against MASLD. Our data indicated that 2 days of exposure to 100 $\:\mathrm{μM}$ vitamin E normalized steatotic status, resulting in reduced *CPT-1* expression in the MASLD model. CPT-1, a key regulator of mitochondrial $\:{\upbeta\:}$-oxidation, is responsible for the transport of long-chain FFAs into the mitochondria. Its upregulation in the MASLD models may reflect an adaptive response to enhanced lipid loading and oxidative stress [33]. Conversely, Tokoro et al. demonstrated that treatment of a diet-induced NAFLD mouse model with 50 mg/kg of vitamin E increased *CPT-1* expression and enhanced $\:\mathrm{{\beta}}$-oxidation, associated with reduced cellular lipid content. Also, they reported that the selected dosage was able to reduce ALT and AST in mice serum [34].

Although CPT-1 is a rate-limiting enzyme of mitochondrial fatty acid $\:\mathrm{{\beta}}$-oxidation, its increased expression in FFAs group may represent a compensatory response to increased fatty acid and stress. Decrease in CPT-1 expression following the vitamin E treatment may reflect the modulation in excessive lipid influx. This may explain the difference between our findings and increase expression reported in other studies [35].

To evaluate the fibrotic features in the MASLD model and the potential modulating effects of vitamin E, expression of *ACTA-2* and *COL1A1* was assessed. Both markers are commonly associated with HSCs activation and pro-fibrotic phenotype. In the present study, FFAs exposure elevated their expressions, suggesting fibrotic features in the MASLD model. Vitamin E treatment particularly reduced the expressions of both markers, indicating potential attenuation of the pro-fibrotic response under MASLD conditions. Consistently, a previous study reported that combination of 20 $\:\mathrm{μM}$ vitamin E and 30 $\:\mathrm{μM}$ hyroxytyrosol showed significant downregulation of *ACTA2* and *COL1A1* in TGF- $\:\mathrm{{\beta}}$-activated LX-2 cells after 24 h of treatment [36]. These results suggest a potential anti-fibrotic effect of vitamin E on MASLD, possibly through attenuation of HSCs-associated fibrotic responses.

Considering lipid metabolism markers, our findings indicate that 100 $\:\mathrm{μM}$ vitamin E improved lipid metabolic homeostasis by modulating expression of key genes involved in fatty acid uptake, lipogenesis, and $\:\mathrm{{\beta}}$-oxidation. The high expression of *SREBP-1c* was observed in the MASLD model and its downregulation by vitamin E treatment. The results were confirmed by a study that demonstrated that 70 mg/kg of vitamin E, for 8 weeks, can downregulate the *SREBP-1c* and other genes involved in the lipogenesis pathway in the NAFLD mice model [22]. The expression of *PPAR*$\:\mathrm{{\gamma}}$ was decreased following the vitamin E treatment in the MASLD model. Likewise, a study by Bartolini et al., indicated that $\:\mathrm{{\alpha}}$-tocopherol (100 $\:\mathrm{μM}\mathrm{,\:1\:day}$) decreased the protein expression of *PPAR*$\:\mathrm{{\gamma}}$ after treatment with 50 $\:\mathrm{μM}$ Oleic acid and 50 $\:\mathrm{μM}$ Palmitic acid on HepG2 monoculture [37]. No significant difference was seen in *PPAR*$\:\mathrm{{\alpha}}$ in our experiment. In contrast, a study on human hepatocyte-derived L02 cells showed *PPAR*$\:\mathrm{{\alpha}}$ downregulation in NAFLD group treated with 100 $\:\mathrm{μM}$ vitamin E [22].

Although therapeutic effects of vitamin E in MASLD have been extensively investigated, most prior studies have relied on monoculture systems or animal models. Our study evaluated these effects within a multicellular co-culture model that incorporates liver-derived ECM, thereby enabling a more physiologically relevant modeling of steatosis, oxidative stress, and cell-cell interactions. The impact of vitamin E was evaluated across multiple levels, from molecular insight to phenotypic outcomes, including hepatic functions, lipid metabolism, and oxidative stress markers. This platform may serve as a reproducible and accessible model for mechanistic investigations and vitamin E-based interventions for MASLD.

Oxidative stress is a central contributor to MASLD pathogenesis, linking lipid accumulation to hepatocellular injury and disease progression. Exposure to FFAs enhanced the generation of ROS, leading to cellular damage, mitochondrial dysfunction, and reduced cell viability [27]. Our results showed that vitamin E treatment in the MASLD model significantly decreased the expression of the *NOX4* gene compared to the FFAs group, suggesting a reduction in ROS levels. Investigation of the effect of *NOX4* expression status in steatosis indicated that *NOX4* modulation improved mitochondrial activity and reduced hepatic inflammation [38]. Moreover, we found that *HO-1* and *NQO1*, downstream targets of Nrf2, were downregulated in the MASLD group and upregulated after vitamin E treatment. Upregulation of *HO-1* has been considered as one of the targets for MASLD treatment [39].

On the other hand, antioxidant markers, including SOD and GSH, were markedly reduced in the MASLD model, indicating a reduction in hepatic antioxidant defense capability. However, vitamin E significantly increased GSH levels, although its effect on SOD was not statistically significant. This suggests that vitamin E may enhance the glutathione system, while its effects on SOD may depend on dosage or treatment duration. Consistently, a study showed that 20 $\:\mathrm{μM}$ of vitamin E modulated oxidative stress by decreasing ROS levels in TGF- $\:\mathrm{{\beta}}$ activated LX-2 cells. They indicated that ROS level was significantly increased in activated LX-2 cells, which would result in suppressing the antioxidant enzymes [36].

At the mechanistic level, the beneficial effects of vitamin E were accompanied by increase in the Nrf2-associated antioxidant responses, suggesting potential involvement of the Nrf2 pathway in the observed protective effects. Protein expression of CES1, a carboxylesterase downstream enzyme of Nrf2, was investigated. This protein is activated by human hepatocyte nuclear factor 4α (HNF-4α), and Nrf2. Activation of this pathway enhanced the expression of antioxidant enzymes and cytoprotective genes, including *HO-1*, *NQO1*, and CES1, thereby reducing ROS levels, lipid accumulation, and inflammatory signals [22, 40, 41]. Decreased expression of CES1 protein in the MASLD model reflects reduced lipid hydrolysis capacity. CES1 is a crucial protein for the hydrolysis of triglycerides and cholesterol esters; thus, its downregulation leads to steatosis and lipid accumulation [22]. Vitamin E significantly reduced lipid content in the MASLD model, as confirmed by Nile Red and Oil Red O staining. Consistently, Chu et al. suggested that treatment of spheroids with fucoidan activated Nrf2 and reduced cellular lipid accumulation [41]. Furthermore, 25 $\:\mathrm{μM}$ of $\:\mathrm{{\alpha}}$-tocopherol significantly reduced lipid content, as confirmed by Oil Red O staining [37]. Vitamin E treatment restored these alterations, leading to reduced oxidative stress and decreased lipid accumulation.

Additionally, FFAs exposure disturbed hepatic functional markers and increased fibrotic signaling, which were partially reversed by vitamin E. Specifically, albumin levels were significantly reduced following FFAs exposure, indicating impaired hepatic protein synthesis due to cellular damage. Vitamin E restored albumin levels, consistent with previous reports linking vitamin E deficiency to MASLD progression [42]. In contrast, AFP levels were elevated in our MASLD model, suggesting hepatic injury and possible activation of proliferative or inflammatory pathways [43]. Furthermore, elevated levels of TGF-$\:\mathrm{{\beta}}$ in the MASLD model indicated activation of LX-2 cells and fibrotic pathways. Our results showed that vitamin E reduced TGF-$\:\mathrm{{\beta}}$ which could be potentially through the inhibition of fibrogenic signaling pathways such as Smad and downregulation of fibrotic-related genes, including *MMP-2* and *COX-2* [44].

Despite the strength of our developed platform in this study, several limitations can be addressed. First, because of the inherent limitations of 2D co-culture models, the system excludes immune components like Kupffer cells/macrophages. Macrophages play a pivotal role in liver inflammation, tissue remodeling, and fibrosis progression, and their integration and non-invasive tracking in liver platforms remain critical for comprehensive disease modeling [45]. Second, while immortalized lines (Huh-7/LX-2) offer a practical starting point, primary human hepatocytes or pluripotent stem cell-derived liver tissues offer superior phenotypic stability and physiological relevance for drug screening platforms [46]. Third, due to the absence of Nrf2 inhibitor, it cannot be conclusively determined that all the observed effects are attributed to the activation of Nrf2 signaling pathway. Future studies incorporating an Nrf2 inhibitor or siRNA-mediated knockdown would provide more reliable evidence regarding the effects of vitamin E on this pathway. Moreover, using various cell populations, primary cells or organoid systems would provide more physiologically relevant insights. Also, implementing various experiments to measure ROS directly would help confidently show the antioxidant effect of vitamin E. Finally, combination of other components or drugs for studying the synergistic effects with vitamin E or using other antioxidants can be considered as potential options.

The present study indicated that vitamin E improved key pathological features of MASLD in a biomimetic 2D co-culture platform. Specifically, vitamin E attenuated oxidative stress, reduced cellular lipid accumulation, and decreased LX-2 cell activation, leading to improved hepatic function. Moreover, these beneficial effects were accompanied by enhanced Nrf2 expression together with upregulation of its downstream genes, suggesting possible link between Nrf2-related antioxidant responses and protective effects of vitamin E.

## Conclusion

MASLD is a multifactorial disorder lacking reliable experimental models for developing therapeutic approaches and modeling molecular pathogenesis. In this study, a biomimetic 2D co-culture system was developed to study the effects of vitamin E treatment in a MASLD model. This model successfully reproduced key pathological features of steatosis phenotype, characterized by lipid accumulation staining and gene and protein expression analysis. Vitamin E treatment enhanced Nrf2-associated antioxidant response, decreased intracellular lipid accumulation, improved hepatocellular functions, and attenuated activation status of LX-2 in the MASLD model. Data suggest that modulation of Nrf2-associated antioxidant pathway may contribute to protective effects of vitamin E. Collectively, these findings support that, in comparison to simple mono-culture platform, this co-culture system provides an available, cost-effective, and more reliable system for studying the cell and molecular mechanisms of MASLD and proposes a potential platform for the first step of drug discovery and introducing candidate medications.

## Supplementary Information

Below is the link to the electronic supplementary material.

Supplementary Material 1

## Data Availability

No datasets were generated or analysed during the current study.

## References

[1] Younossi ZM, Kalligeros M, Henry L (2024) Epidemiology of metabolic dysfunction-associated steatotic liver disease. Clin Mol Hepatol 31(Suppl):S32 39159948 10.3350/cmh.2024.0431 PMC11925440

[2] Carnicero JD et al (2025) Clinical and economic burden of metabolic dysfunction-associated steatotic liver disease (MASLD) in a Spanish mediterranean region: a population-based study. J Clin Med 14(7):2441 40217891 10.3390/jcm14072441 PMC11989979

[3] Chan W-K et al (2023) Metabolic Dysfunction-Associated Steatotic Liver Disease (MASLD): A State-of-the-Art Review. J Obes Metabolic Syndrome 32(3):197–213 10.7570/jomes23052 PMC10583766 37700494

[4] Xu M et al (2024) A multidrug donor preconditioning improves steatotic rat liver allograft function and recipient survival after transplantation. Transpl Int 37:13557 39726675 10.3389/ti.2024.13557 PMC11671227

[5] Vrentzos E et al (2025) Nutraceutical strategies for metabolic dysfunction-associated steatotic liver disease (MASLD): A path to liver health. Nutrients 17(10):1657 40431398 10.3390/nu17101657 PMC12113997

[6] Alwahsh SM et al (2025) Excessive intake of fructose and alcohol aggravates high-fat diet-induced steatohepatitis and changes hepatic iron homeostasis. Histochem Cell Biol 163(1):77 40745231 10.1007/s00418-025-02402-4

[7] Abdelmalek MF et al (2010) Increased fructose consumption is associated with fibrosis severity in patients with nonalcoholic fatty liver disease. Hepatology 51(6):1961–1971 20301112 10.1002/hep.23535 PMC2922495

[8] Ishimoto T et al (2013) High-fat and high‐sucrose (western) diet induces steatohepatitis that is dependent on fructokinase. Hepatology 58(5):1632–1643 23813872 10.1002/hep.26594 PMC3894259

[9] Ichimura M et al (2015) High-fat and high‐cholesterol diet rapidly induces non‐alcoholic steatohepatitis with advanced fibrosis in S prague–D awley rats. Hepatol Res 45(4):458–469 24827559 10.1111/hepr.12358

[10] Yang Z et al (2025) MASLD development: From molecular pathogenesis toward therapeutic strategies. Chin Med J 138(15):1807–1824 40640088 10.1097/CM9.0000000000003629 PMC12321477

[11] Zhao K et al (2026) Adipokines regulate the development and progression of MASLD through organellar oxidative stress. Hepatol Commun 10(2):e0639 10.1097/HC9.0000000000000639 PMC11781772 39878681

[12] Termite F et al (2025) Gut microbiota at the crossroad of hepatic oxidative stress and MASLD. Antioxidants 14(1):56 39857390 10.3390/antiox14010056 PMC11759774

[13] Arroyave-Ospina JC et al (2021) Role of oxidative stress in the pathogenesis of non-alcoholic fatty liver disease: implications for prevention and therapy. Antioxidants 10(2):174 33530432 10.3390/antiox10020174 PMC7911109

[14] Jakubek P et al (2024) Oxidative stress in metabolic dysfunction-associated steatotic liver disease (MASLD): How does the animal model resemble human disease? FASEB J 38(3):e23466 38318780 10.1096/fj.202302447R

[15] Perumpail BJ et al (2018) The role of Vitamin E in the treatment of NAFLD. Diseases 6(4):86 30249972 10.3390/diseases6040086 PMC6313719

[16] Svobodová G et al (2025) Metabolic dysfunction-associated steatotic liver disease-induced changes in the antioxidant system: A review. Arch Toxicol 99(1):1–22 39443317 10.1007/s00204-024-03889-x PMC11748479

[17] Suzuki T, Takahashi J, Yamamoto M (2023) Molecular basis of the KEAP1-NRF2 signaling pathway. Mol Cells 46(3):133–141 36994473 10.14348/molcells.2023.0028 PMC10070164

[18] He F, Ru X, Wen T (2020) NRF2, a transcription factor for stress response and beyond. Int J Mol Sci 21(13):4777 32640524 10.3390/ijms21134777 PMC7369905

[19] Fu Y et al (2024) Progress in the Study of Animal Models of Metabolic Dysfunction-Associated Steatotic Liver Disease. Nutrients 16:3120 10.3390/nu16183120 PMC11435380 39339720

[20] Huang Z et al (2025) Three-Dimensional Dynamic Cell Models for Metabolic Dysfunction-Associated Steatotic Liver Disease Progression. BME Front 6:0181 41035773 10.34133/bmef.0181 PMC12480745

[21] Li M et al (2024) Metabolic dysfunction-associated steatotic liver disease in a dish: human precision-cut liver slices as a platform for drug screening and interventions. Nutrients 16(5):626 38474754 10.3390/nu16050626 PMC10934612

[22] He W et al (2019) Vitamin E ameliorates lipid metabolism in mice with nonalcoholic fatty liver disease via Nrf2/CES1 signaling pathway. Dig Dis Sci 64(11):3182–3191 31076985 10.1007/s10620-019-05657-9

[23] Saheli M et al (2018) Three-dimensional liver‐derived extracellular matrix hydrogel promotes liver organoids function. J Cell Biochem 119(6):4320–4333 29247536 10.1002/jcb.26622

[24] Alevra Sarika N et al (2020) Human liver-derived extracellular matrix for the culture of distinct human primary liver cells. Cells 9(6):1357. 10.3390/cells9061357 32486126 PMC7349413

[25] Ma Y et al (2023) Three-dimensional cell co-culture liver models and their applications in pharmaceutical research. Int J Mol Sci 24(7):6248 37047220 10.3390/ijms24076248 PMC10094553

[26] Asadollahi N et al (2024) Bioengineering scalable and drug-responsive in vitro human multicellular non-alcoholic fatty liver disease microtissues encapsulated in the liver extracellular matrix-derived hydrogel. EXCLI J 23:421 38741724 10.17179/excli2023-6878 PMC11089098

[27] Majidi F et al (2025) Bio-fabrication of fibrotic liver microtissues as a scalable and drug-responsive in vitro model. Int J Pharm, 30(685):126242 10.1016/j.ijpharm.2025.126242 41052739

[28] Palasantzas VE et al (2026) Assessing Nutraceuticals for Hepatic Steatosis: A Standardized In Vitro Approach. Nutrients 18(3):388 41683214 10.3390/nu18030388 PMC12899271

[29] Podszun MC, Frank J (2021) Impact of vitamin E on redox biomarkers in non-alcoholic fatty liver disease. Redox Biol 42:101937 33773953 10.1016/j.redox.2021.101937 PMC8113042

[30] Yao H-R et al (2011) Lipotoxicity in HepG2 cells triggered by free fatty acids. Am J translational Res 3(3):284 PMC3102573 21654881

[31] Soret P-A et al (2020) In vitro and in vivo models of non-alcoholic fatty liver disease: a critical appraisal. J Clin Med 10(1):36 33374435 10.3390/jcm10010036 PMC7794936

[32] Tutty MA, Movia D, Prina-Mello A (2022) Three-dimensional (3D) liver cell models-a tool for bridging the gap between animal studies and clinical trials when screening liver accumulation and toxicity of nanobiomaterials. Drug Delivery Translational Res 12(9):2048–2074 10.1007/s13346-022-01147-0 PMC9066991 35507131

[33] AlNafea HM, Korish AA (2021) Activation of the Peroxisome Proliferator-Activated Receptors (PPAR‐α/γ) and the Fatty Acid Metabolizing Enzyme Protein CPT1A by Camel Milk Treatment Counteracts the High‐Fat Diet‐Induced Nonalcoholic Fatty Liver Disease. PPAR Res 2021(1):5558731 34306045 10.1155/2021/5558731 PMC8285205

[34] Tokoro M et al (2021) α-Tocopherol suppresses hepatic steatosis by increasing CPT‐1 expression in a mouse model of diet‐induced nonalcoholic fatty liver disease. Obes Sci Pract 7(1):91–99 33680496 10.1002/osp4.460 PMC7909598

[35] Fondevila MF et al (2022) Inhibition of carnitine palmitoyltransferase 1A in hepatic stellate cells protects against fibrosis. J Hepatol 77(1):15–28 35167910 10.1016/j.jhep.2022.02.003

[36] Panera N et al (2022) Combination treatment with hydroxytyrosol and Vitamin E improves NAFLD-related fibrosis. Nutrients, 14(18):3791 10.3390/nu14183791 PMC9505330 36145170

[37] Bartolini D et al (2017) Nonalcoholic fatty liver disease impairs the cytochrome P-450-dependent metabolism of α-tocopherol (vitamin E). J Nutr Biochem 47:120–131 28628909 10.1016/j.jnutbio.2017.06.003

[38] Park JS, Rustamov N, Roh YS (2023) The Roles of NFR2-Regulated Oxidative Stress and Mitochondrial Quality Control in Chronic Liver Diseases. Antioxid (Basel), 12(11):1928 10.3390/antiox12111928 PMC10669501 38001781

[39] Li N et al (2024) The NRF-2/HO-1 Signaling Pathway: A Promising Therapeutic Target for Metabolic Dysfunction-Associated Steatotic Liver Disease. J Inflamm Res 17:8061–8083 39512865 10.2147/JIR.S490418 PMC11542495

[40] Shiragannavar VD et al (2023) The ameliorating effect of withaferin A on high-fat diet-induced non-alcoholic fatty liver disease by acting as an LXR/FXR dual receptor activator. Front Pharmacol 14:1135952 36909161 10.3389/fphar.2023.1135952 PMC9995434

[41] Chu X et al (2025) Fucoidan ameliorates lipid accumulation, oxidative stress, and NF-κB-mediated inflammation by regulating the PI3K/AKT/Nrf2 signaling pathway in a free fatty acid-induced NAFLD spheroid model. Lipids Health Dis 24(1):55 39962463 10.1186/s12944-025-02483-z PMC11831825

[42] Tsou P, Wu CJ (2019) Serum Vitamin E Levels of Adults with Nonalcoholic Fatty Liver Disease: An Inverse Relationship with All-Cause Mortality in Non-Diabetic but Not in Pre-Diabetic or Diabetic Subjects. J Clin Med, 8(7):1057 10.3390/jcm8071057 PMC6678235 31330971

[43] Wang Y et al (2023) Elevated Serum Alpha-fetoprotein Levels in Non-alcoholic Steatohepatitis: Possible Molecular Mechanisms and Potential Clinical Significance. Gene Expr 22(2):135–140

[44] Sumida Y et al (2021) Role of vitamin E in the treatment of non-alcoholic steatohepatitis. Free Radic Biol Med 177:391–403 34715296 10.1016/j.freeradbiomed.2021.10.017

[45] Sharkey J et al (2017) Functionalized superparamagnetic iron oxide nanoparticles provide highly efficient iron-labeling in macrophages for magnetic resonance–based detection in vivo. Cytotherapy 19(4):555–569 28214127 10.1016/j.jcyt.2017.01.003 PMC5357746

[46] Alwahsh SM, Rashidi H, Hay DC (2018) Liver cell therapy: is this the end of the beginning? Cell Mol Life Sci 75(8):1307–1324 29181772 10.1007/s00018-017-2713-8 PMC5852182

