# Supplementary material for: Vitamin E treatment modulates steatotic and attenuates oxidative stress in a MASLD in vitro model
Source: Mol Biol Rep. 2026 Sep 7;53(1):1543. doi: 10.1007/s11033-026-12691-0 (PMC13550042; doi:10.1007/s11033-026-12691-0)
Supplement: Supplementary file 1 [file 11033_2026_12691_MOESM1_ESM.docx]

**Table S1. Primer sequences used in qPCR assessment.**

| **Gene** | **Forward** | **Reverse** |
| --- | --- | --- |
| *HO-1* | ATGGCCTCCCTGTACCACATC | TGTTGCGCTCAATCTCCTCCT |
| *NQO-1* | CGCAGACCTTGTGATATTCCAG | CGTTTCTTCCATCCTTCCAGG |
| *SREBP-1c* | CGGAACCATCTTGGCAACAGT | CGCTTCTCAATGGCGTTGT |
| *CD36* | CTTTGGCTTAATGAGACTGGGAC | GCAACAAACATCACCACACCA |
| *PPAR*$\gamma$ | CTAAAGAGCCTGCGAAAG | TGTCTGTCTCCGTCTTCTTG |
| *PPAR*$\alpha$ | CTGCTATAATTTGCTGTGGAGA | TTGGGAAGAGAAAGGTATCATC |
| *NOX4* | ATTTCGGTCATAAGTCATCCCTC | AATCGTTCTGTCCAGTCTCCT |
| *CPT-1* | ATCAATCGGACTCTGGAAACGG | TCAGGGAGTAGCGCATGGT |
| *GAPDH* | GAAATCCCATCACCATCTTCC | GGCTGTTGTCATACTTCTCAT |
| *ACTA2* | TGGCTATTCCTTCGTTACTACTGCT | CATCAGGCAACTCGTAACTCTTCTC |
| *COL1A1* | CCTGTCTGCTTCCTGTAAAC | ATGTTCGGTTGGTCAAAGATAAA |

**Table S2. Commercial kits used in assessments.**

| **ELISA marker** | **Kit** |
| --- | --- |
| Albumin | ab108788-Albumin Human ELISA Kit |
| $\alpha$-AFP | ab108838-Human alpha Fetoprotein ELISA Kit |
| Glutathione | Glutathione Assay Kit CS0260 Sigma-Aldrich |
| SOD | ab314351- Human Superoxide Dismutase 1 ELISA Kit |
| TGF-$\beta$ | Human TGF-$\beta$ DY240-05 DuoSet ELISA |

**Fig S1. Supplementary. Vehicle (EtOH 95%). A)** The Oil Red O staining of control, FFAs, vehicle, and vitamin E indicated no significant difference between FFAs and vehicle, which suggested that the vehicle did not have any effect on lipid accumulation, and all the positive impact are according to vitamin E effects. **B)** Also, the quantification of this staining confirms this hypothesis.
